# Sertoliform endometrioid carcinoma of the right ovary

**DOI:** 10.1530/EDM-23-0046

**Published:** 2023-09-28

**Authors:** Mohammad Alali, Sulaiman Hajji, Khalid Aljenaee

**Affiliations:** 1 Kuwait Board of Internal Medicine, Kuwait City, Safat, Kuwait; 2 Department of Internal Medicine, Adan Hospital, Kuwait City, Kuwait

**Keywords:** Adult, Female, Other, Kuwait, Ovaries, Developmental endocrinology, Endocrine-related cancer, Gynaecological endocrinology, Endometrial cancer, Unique/unexpected symptoms or presentations of a disease, September, 2023

## Abstract

**Summary:**

Endometrioid carcinomas of the ovary are a subtype of epithelial ovarian tumors, with sertoliform endometrioid carcinomas being a rare variant. We report a case of a previously healthy premenopausal woman presenting with androgenic symptoms in the form of hirsutism and male pattern alopecia. On further testing, she was found to have high levels of luteinizing hormone and total testosterone levels, and imaging revealed a large pelvic abdominal mass in the right ovary. She underwent total hysterectomy with bilateral salpingo-oophorectomy. Microscopy and histopathology confirmed the diagnosis of sertoliform endometrioid carcinoma. Her symptoms improved significantly on follow-up. Androgenic tumors might not be common in premenopausal women; however, it is important to maintain a high level of suspicion in patients presenting with virilizing symptoms especially of rapid progression.

**Learning points:**

- Our 47-year-old patient presented with virilizing symptoms that were rapidly progressing, which raises the suspicion of an underlying androgen secreting neoplasm.
- Sertoliform endometrioid carcinoma (SEC) is an extremely rare variant of endometrioid carcinomas and tend to present at an earlier stage as compared to most endometrioid carcinomas of the ovary.
- Recognition of SEC in virilizing patients is important as it is a well-differentiated, low-grade malignancy with a good prognosis when confined to the ovary.

## Background

We report a 47-year-old premenopausal lady presenting to our clinic with virilizing symptoms, and on further evaluation she was diagnosed with sertoliform endometrioid carcinoma (SEC) of the right ovary. Endometrioid carcinomas of the ovary are a subtype of epithelial ovarian tumors. The vast majority are malignant and invasive. On imaging, they are usually characterized as complex and nonspecific solid-cystic masses found associated with endometriosis. However, endometrioid carcinoma of the ovary resembling sex cord stromal tumors is a rare variant of endometrioid adenocarcinomas that focally looks like a sex cord stromal tumor with Sertoli, Leydig, or granulosa cells. SEC of the ovary is one variant that bears histologic similarity to Sertoli and Sertoli–Leydig cell tumor (SLT).

## Case presentation

A 47-year-old healthy woman, who had not yet reached menopause, visited the endocrine clinic due to increased hirsutism that had developed rapidly over the past 8 months. The excessive hair growth was primarily over her face, chest, lower abdomen, inner thighs, and upper back. In addition, she experienced thinning and loss of hair on her scalp but did not suffer from acne vulgaris. Her menstrual cycles were regular every 23 to 27 days with no abnormal vaginal bleeding. She also complained of chronic constipation and abdominal distension for the past 3–4 months, but she did not seek any medical attention.

The patient had no significant medical or family history, did not take any medications regularly, and denied the use of herbal remedies. Physical examination indicated normal vital signs but revealed significant hirsutism with a modified Ferriman–Gallway score of 17/36. Examination of her abdomen showed a palpable mass in the lower abdomen, measuring 25 cm, which was rounded and hard, but no change in the skin color overlaying it.

## Investigation

**Figure 1:**
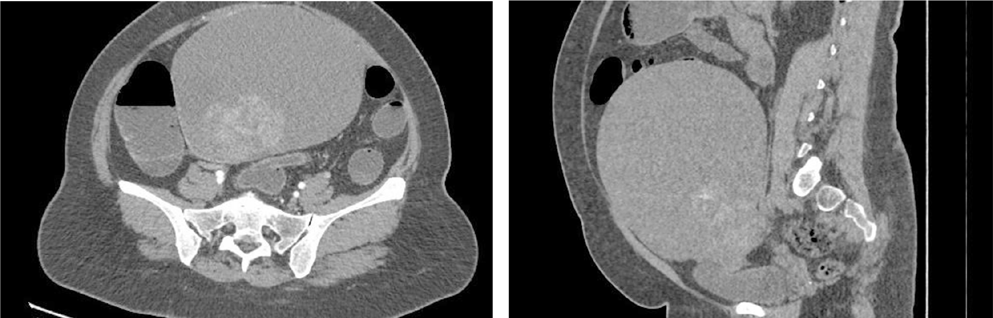
CT abdomen and pelvis.

Laboratory tests showed FSH at 9.96 IU/L (reference range (RR): 1–18 IU/L), LH at 15.4 IU/L (RR: 1.8–8.6 IU/L), elevated total testosterone at 4.38 nmol/L (RR: 0.5–2.4 nmol/L), and DHEAS at 161 μc/dL (RR: 44.7– 376.7 μg/dL), but normal levels of estradiol and SHBG (see Table 1). A CT scan of her abdomen and pelvis revealed a large pelvic abdominal mass (measuring 21 × 20.5 × 17 cm) that consisted of cystic and basal soft tissue components related to the right ovary (see Fig. 1).

**Table 1:** Hormonal profile.

Laboratory tests	Preoperative	3 months postoperative	Reference range
LH (IU/L)	15.4	13.2	1.8–8.6
FSH (IU/L)	9.96	8.2	1–18
Total testosterone (nmol/L)	4.38	0.6	0.5–2.4
DEA-S (μg/dL)	161	137.5	44.7–376.7

FSH, follicle-stimulating hormone; LH, luteinizing hormone.

## Treatment

Subsequently the patient was referred for surgery and total hysterectomy with bilateral salpingo-oophorectomy was done, the right ovary was grossly enlarged (measuring 9.5 × 8 × 4.8 cm) and was seen to be replaced with a solid cystic tumor and ruptured capsule. Microscopic examination revealed that it consisted of round-to-solid tubules lined by pseudostratified columnar epithelium, resembling a Sertoli tumor, along with a conventional endometrioid tumor.

A histopathologic diagnosis of SEC was made. Immunohistochemistry showed tumor cells strongly immunoreactive for epithelial membrane antigen cytokeratin but negative for inhibin, so confirming the diagnosis of SEC of the right ovary. The tumor was in stage IA according to FIGO (International Federation of Gynecology and Obstetrics) staging system, as the cancer was limited to the ovary without any evidence of spread to nearby lymph nodes or other organs.

## Outcome and follow-up

Three months postoperatively her androgenic symptoms started to fade already and androgen levels improved: total testosterone dropped to 0.6 nmol/L and DHEAS 137.5 μg/dL (Table 1). On follow-up, her symptoms improved dramatically over 3–6 months.

## Discussion

**Figure 2:**
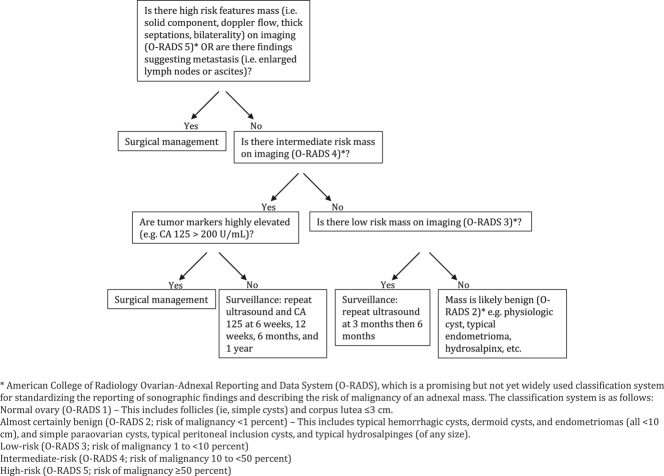
Approach to a premenopausal patient with an adnexal mass.

Androgen-secreting tumors are not commonly found in premenopausal women with hirsutism, accounting for only 0.2% of cases, but a significant 50% of them are malignant upon diagnosis (1). Various types of androgen-secreting ovarian tumors exist, including steroid cell tumors, Leydig tumors, granulosa cell tumors, Sertoli cell tumors, SLT, gonadoblastomas, ovarian metastases from neuroendocrine tumors, and other rare forms (2). Table 2 outlines some of their comparative features (3, 4, 5). SEC is an extremely rare variant that usually affects older patients compared to SLT, with SLT patients having more evident virilizing symptoms (6). Virilizing symptoms experienced by patients include hirsutism, male pattern alopecia, voice deepening, clitoromegaly, acne, breast atrophy, oligomenorrhea, or amenorrhea. Gross appearance of SEC tumors is predominantly solid, with our patient’s tumor being solid with cystic components. Microscopic appearance shows anastomosing cords, trabeculae, nests, or tubules lined by pseudostratified columnar epithelium with elongated nuclei resembling SLTs. The presence of areas with the usual pattern of endometrioid carcinoma and the presence of mucin at the apical borders of the tumor cells usually support a diagnosis of SEC (6). Immunohistochemistry is the most reliable technique to distinguish SEC from SLTs and confirm the diagnosis. Cytokeratin and epithelial membrane antigen are typically expressed by SEC, while, on the other hand, almost all SLTs express Inhibin (7). Treating SEC, as with other epithelial ovarian cancers, mainly involves debulking surgery and chemotherapy if indicated. Figure 2 outlines an approach to a premenopausal woman presenting with an adnexal mass (after confirmation by conducting a thorough history, pelvic examination, and pelvic imaging with ultrasound, CT scan, or MRI) (8, 9, 10, 11, 12).

**Table 2:** Comparison of findings in hyperandrogenic ovarian tumors.

Tumor	Age at presentation	Characteristic features of the ovarian mass	Hormonal effects	Other potential tumor markers	Behavior
Granulosa cell tumor	Adult subtype occurs most commonly in middle-aged and older women (median age 50 to 54 years) and comprises 95% of these neoplasms. Juvenile subtype typically develops before puberty, and comprises 5% of all granulosa cell tumors.	Gross: Appearance is variable; usually large (mean diameter 12 cm), tan or yellow, and either soft or firm. U/S: Usually unilateral, echogenic, septated cystic, or solid mass. Ascites may be present.	Signs/symptoms of excess estrogen in over one-half of patients. Endometrial hyperplasia/intraepithelial neoplasia in 25–50% and endometrial carcinoma in 5–10%. The endometrial adenocarcinomas are usually early stage and well differentiated. Virilization is possible, but rare.	Inhibin AMH CA 125	Malignant
Sertoli–Leydig cell tumor	Usually in the second and third decades of life. Approximately 75% occur in women under age 40 years (mean age at diagnosis is 25), but they occur in all age groups	Gross: Yellow and lobulated, with a smooth external surface. U/S: Often large (average 16 cm in maximal diameter). Most are unilateral and solid but may contain areas of closely packed small cysts.	At least one-third of patients have virilization. Less than one-third of patients have signs/symptoms of excess estrogen.	AFP Inhibin CA 125	Benign or malignant
Sertoli cell tumor	Usually women of reproductive age but may occur in children as young as age 2 and postmenopausal women	Gross: Yellow to brown. U/S: Usually unilateral, solid, but may have several cystic areas.	Approximately one-half produce functional hormones. Most commonly signs/symptoms of excess estrogen, but virilization can occur. Rarely both estrogens and androgens are produced.	Renin	Benign or malignant
Luteinized thecoma associated with sclerosing peritonitis	Broad age range	Gross: Surface may be smooth, polypoid, lobulated, or cerebriform. U/S: Bilateral and solid. Massive ascites is common.	Hormonal manifestations are usually absent. Rare cases associated with estrogen or androgen production.	NA	Benign. However, it can be associated with significant morbidity and occasional death due to severe adhesive disease and bowel obstruction.

## Declaration of interest

The authors declare that there is no conflict of interest that could be perceived as prejudicing the impartiality of the research reported.

## Funding

This research did not receive any specific grant from any funding agency in the public, commercial or not-for-profit sector.

## Patient consent

Written informed consent for publication of their clinical details and clinical images was obtained from the patient.

## Author contribution statement

Mohammad Alali: treating physician, drafted the article; Sulaiman Hajji: revised the article; and Khalid Aljenaee: treating consultant, revised the article.
